# Treatment of bleeding from a portion of pancreatojejunostomy after pancreaticoduodenectomy with division of the splenic vein: two case reports

**DOI:** 10.1186/s40792-019-0687-5

**Published:** 2019-08-08

**Authors:** Hiroki Kushiya, Takehiro Noji, Daisuke Abo, Takeshi Soyama, Kimitaka Tanaka, Yoshitsugu Nakanishi, Toshimichi Asano, Toru Nakamura, Takahiro Tsuchikawa, Keisuke Okamura, Satoshi Hirano

**Affiliations:** 1 Department of Gastroenterological Surgery 2, Hokkaido University Faculty of Medicine, Kita 15 Nishi 7, Kita-ku, Sapporo, Hokkaido 060-8638 Japan; 2 Department of Diagnostic and Interventional Radiology, Hokkaido University Hospital, Kita 15 Nishi 7, Kita-ku, Sapporo, 060-8638 Japan

**Keywords:** Pancreaticoduodenectomy, Left-sided portal hypertension, Interventional radiology, Intestinal bleeding

## Abstract

**Background:**

There is no definitive strategy for gastrointestinal bleeding due to left-sided portal hypertension after pancreaticoduodenectomy (PD) for pancreatic cancer (PC) with concomitant portal vein resection (PVR).

**Case presentation:**

Case 1: A 70-year-old woman underwent a PD for PC with PVR. Seven years after her surgery, she suffered severe anemia with suspected gastrointestinal bleeding. Computed tomography scan (CT) revealed varices at a portion of the pancreaticojejunostomy (PJ). Angiography revealed that splenic venous flow drained into the varices and then into the portal vein. A diagnosis of bleeding varices of the PJ due to left-sided portal hypertension was made. Following a partial splenic artery embolization, her anemia improved.

Case 2: An 80-year-old male underwent a PD for pancreatic head cancer combined with resection of the confluence of the portal and splenic veins with a reconstruction between the portal and superior mesenteric veins. Eighteen months after his surgery, he developed melena with negative endoscopy findings in his large and small bowel. CT revealed varices at the site of the PJ that communicated with the jejunal and portal veins. He underwent obliteration of the varices via a trans-portal-venous approach. As a result, he remained without melena until he died of PC 17 months after the embolization.

**Conclusions:**

Left-sided portal hypertension following a PD with bleeding varices can be treated by interventional radiology with minimal invasiveness.

## Background

Left-sided portal hypertension (LSPH) is a localized form of portal hypertension that occurs as a result of an isolated thrombosis or obstruction of the splenic vein (SV) [1]. LSPH is different from PH in terms of normal liver function and no obstruction of the portal vein (PV) [2]. Also, it has been reported that LSPH occurred after pancreaticoduodenectomies (PDs) without SV reconstructions [3, 4]. Herein, we discuss two different types of interventional radiology (IVR) methods for treating intestinal bleeding from pancreaticojejunostomies (PJs) due to LSPH after PDs with concomitant PV resections (PVRs).

## Case presentation

### Case 1

**Fig. 1:**
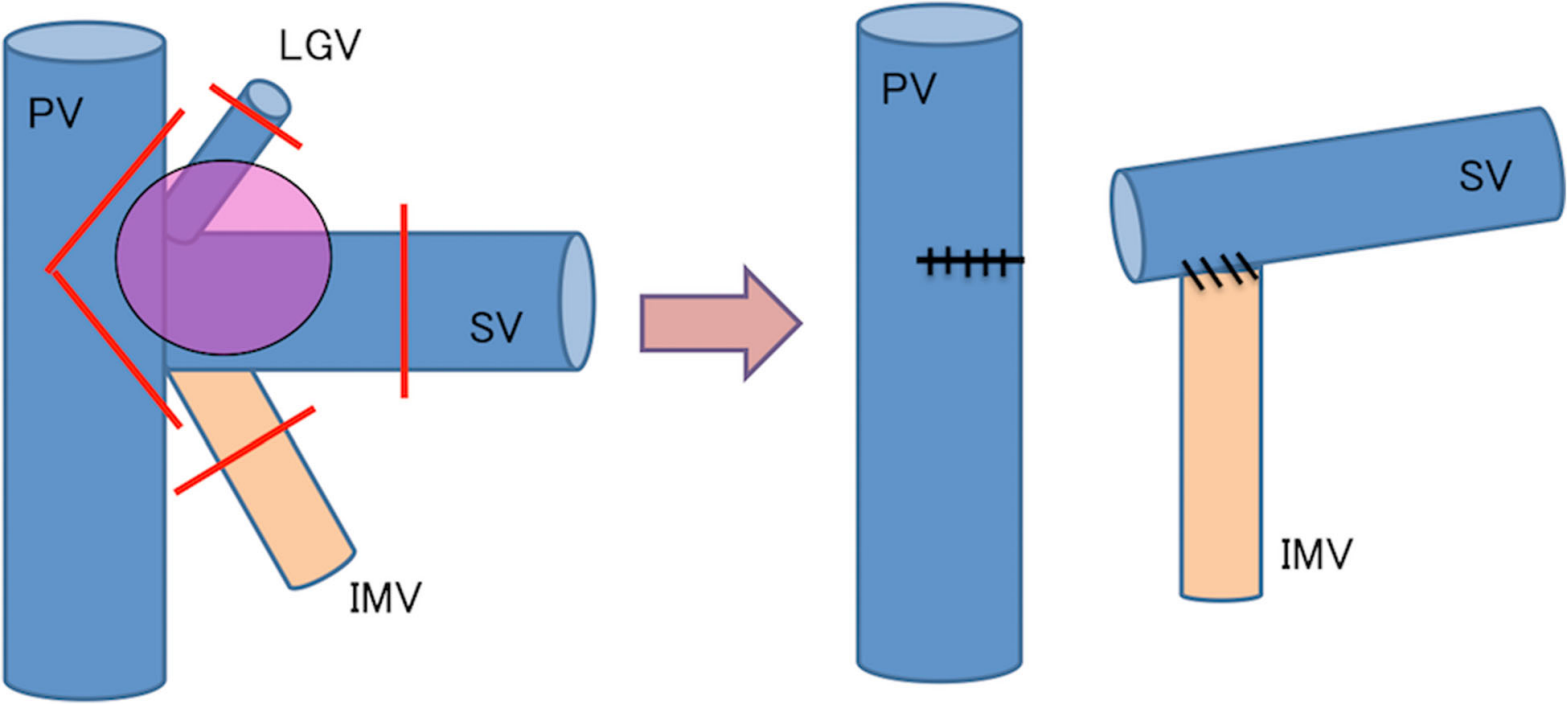
Pancreaticoduodenectomy combined with a concomitant resection of the confluence of the PV and SV. Side-to-end anastomosis was performed between the SV and IMV. *PV* portal vein, *LGV* left gastric vein, *SV* splenic vein, *IMV* inferior mesenteric vein

**Fig. 2:**
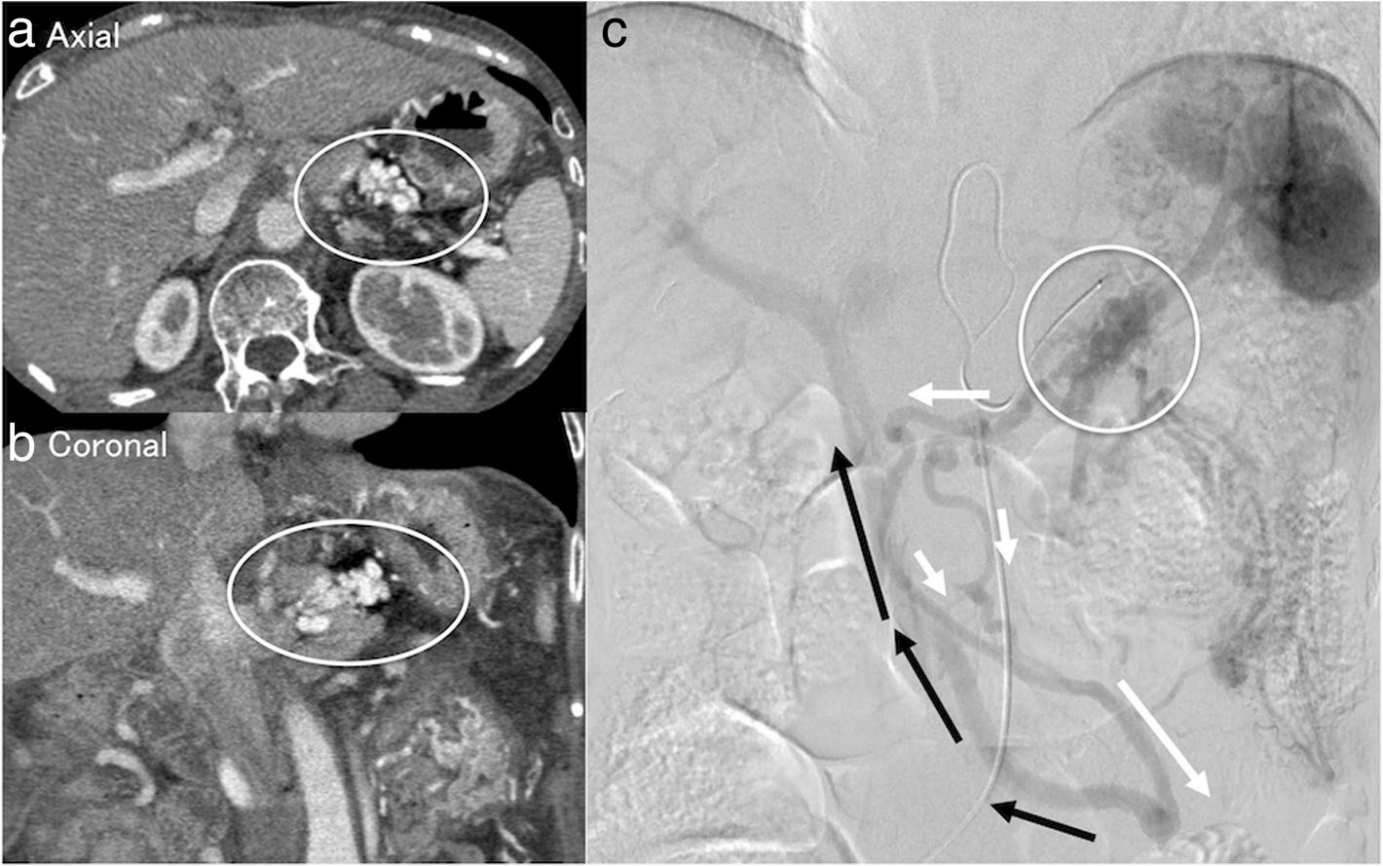
**a**, **b** Computed tomography image showing pancreaticojejunostomy varices. **c** Image of digital subtraction arterial portography from the splenic artery. The flow of splenic vein drain into the varices around the pancreaticojejunostomy (white circle) and finally toward the superior mesenteric vein (black arrow) via the elevated jejunal vein (white arrow)

A 70-year-old woman underwent PD for pancreatic cancer (PC) with a concomitant PVR. She also underwent an SV and inferior mesenteric vein (IMV) resection. The side-to-end anastomosis was performed only between the SV and IMV (Fig. 1). She discharged without complications. Seven years after her surgery, she suffered severe anemia and gastrointestinal bleeding was suspected. However, we could not find a bleeding lesion using gastrointestinal endoscopes. Computed tomography (CT) revealed varices at a portion of the PJ (Fig. 2a, b). Angiography revealed that splenic venous flow drained into the varices around the PJ and then ran into the PV (Fig. 2c). Therefore, we diagnosed that her anemia originated from varices at the PJ with LSPH.

**Fig. 3:**
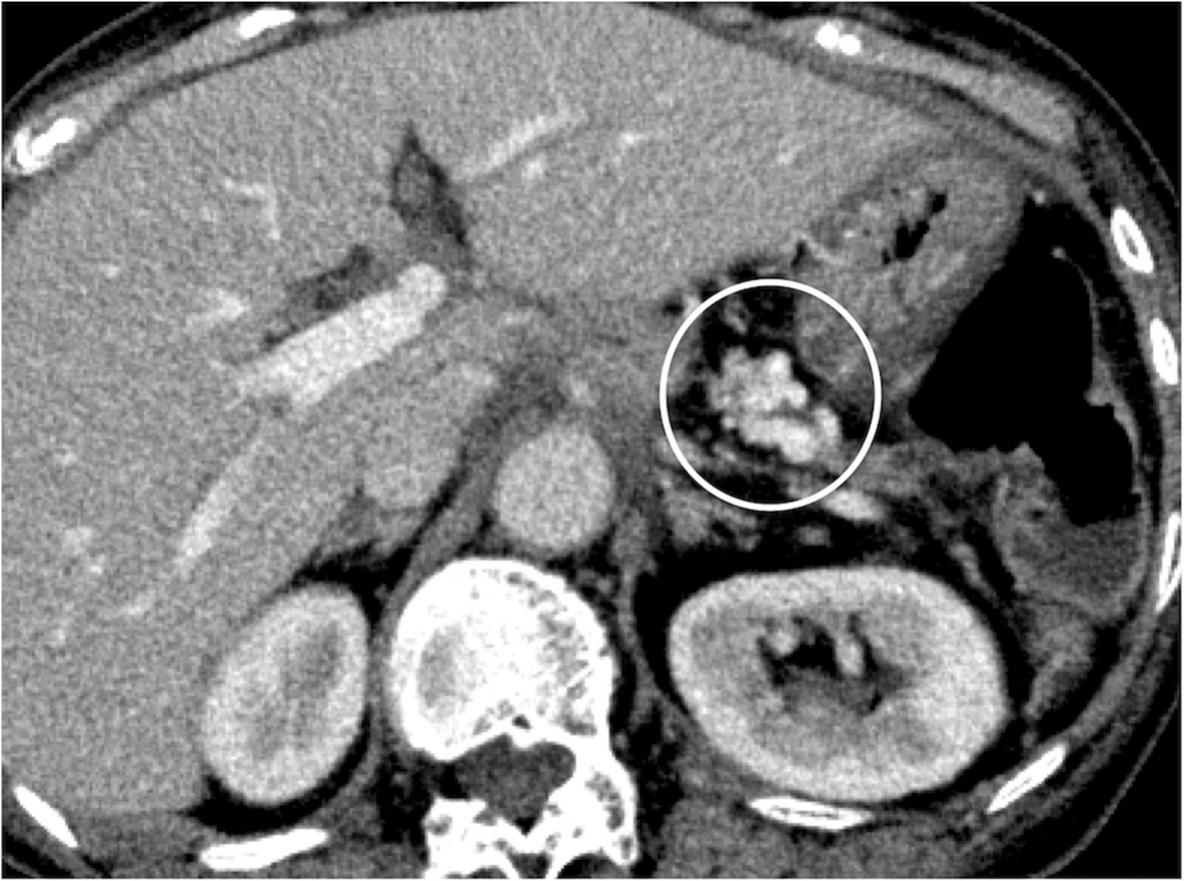
Pancreaticojejunostomy varices remained by follow-up CT at 3 years after first stage PSE

We selected partial splenic artery embolization (PSE) for hemostasis. At the first stage, we embolized 70% of the splenic arterial flow. Ten months after the first stage PSE, she again complained of melena and anemia, for which we performed a second stage PSE (embolized 90% of splenic artery flow). Although follow-up CT at 3 years after first stage PSE showed the varices around the PJ (Fig. 3), her bleeding episodes have not been observed during a 2-year follow-up period.

### Case 2

**Fig. 4:**
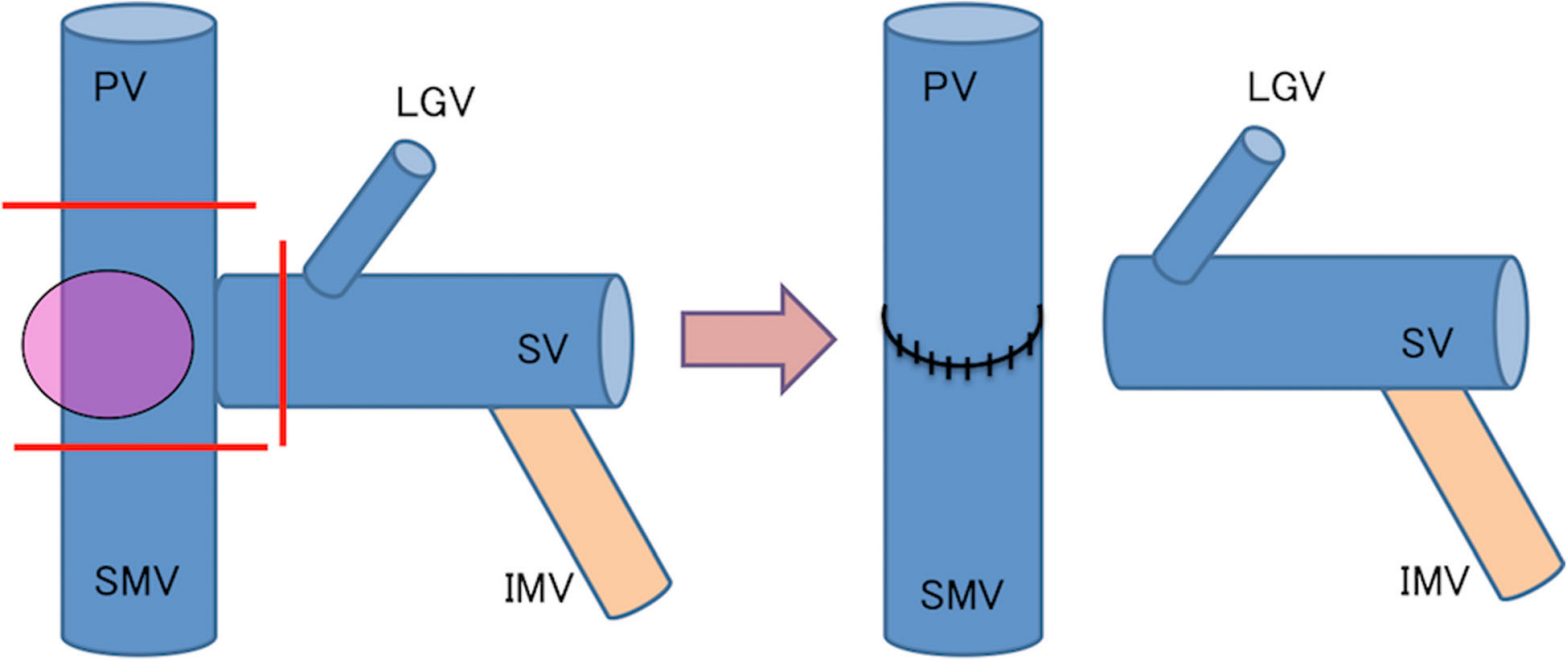
Pancreaticoduodenectomy combined with concomitant resection of the confluence of the PV and SV. Reconstruction was performed only between the PV and SMV: the IMV was preserved. *PV* portal vein, *LGV* left gastric vein, *SV* splenic vein, *IMV* inferior mesenteric vein, *SMV* superior mesenteric vein

**Fig. 5:**
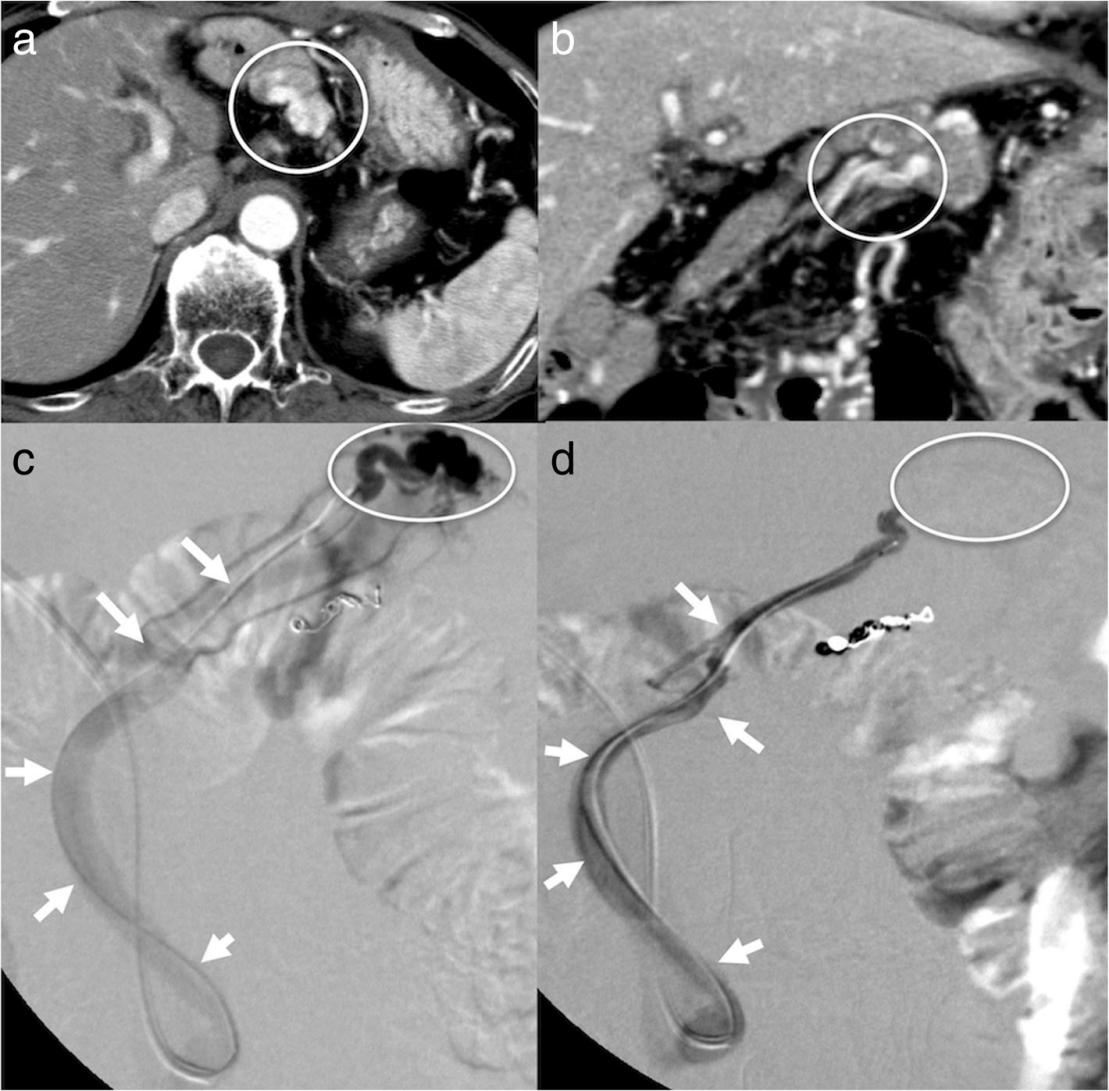
**a**, **b** Computed tomography image showing varices at the pancreaticojejunostomy that communicated with the elevated jejunal and portal veins. **c**, **d** Image of retrograde trans-portal-venous obliteration (white arrow is the elevated jejunal vein). Retrograde venography before obliteration (**c**) showing the varices around the pancreaticojejunostomy (white circle). Retrograde venography after obliteration (**d**) showing that the varices around the pancreaticojejunostomy disappear (white circle)

**Fig. 6:**
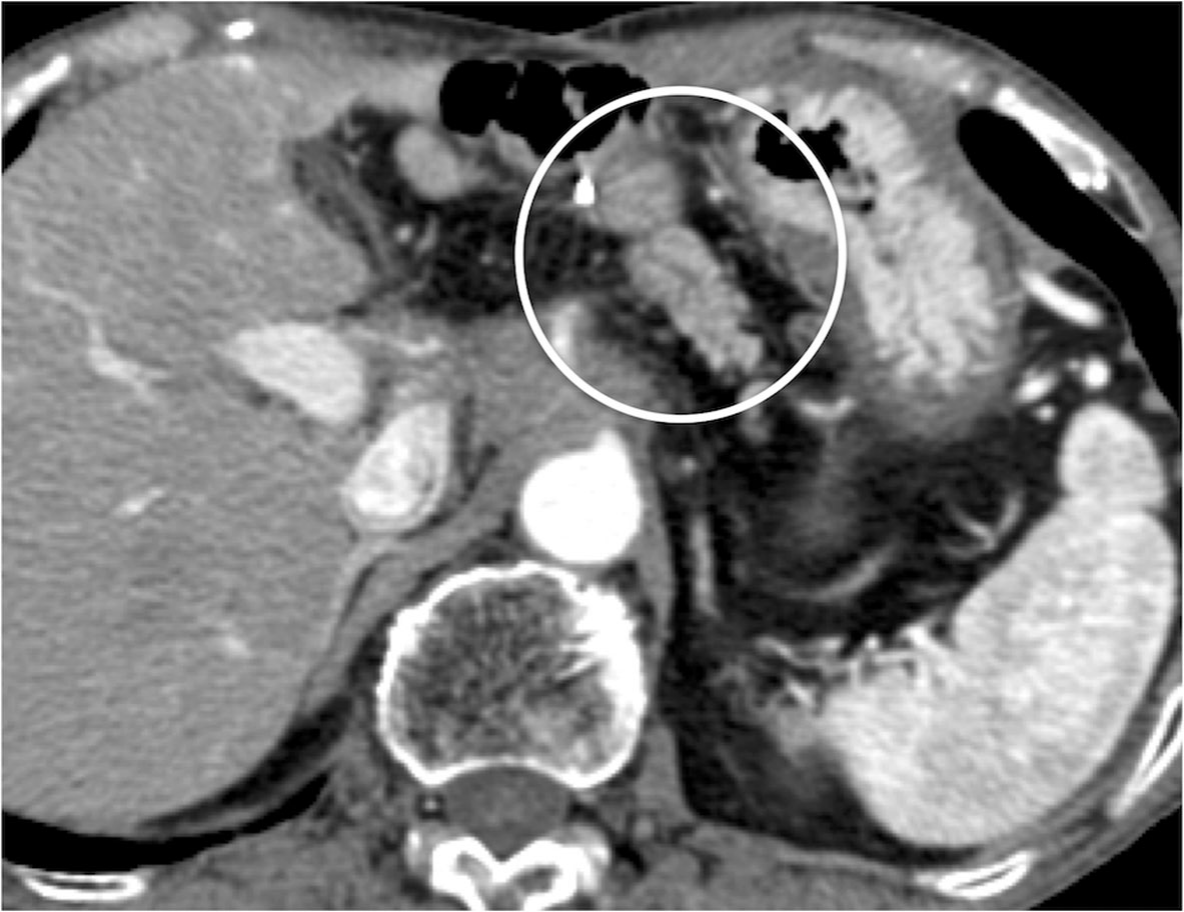
Follow-up CT at 1 year after procedure showed no varices at a portion of the PJ

An 80-year-old male underwent a PD for pancreatic head cancer combined with resection of the confluence of the PV and SV in addition to a reconstruction between the PV and superior mesenteric vein (SMV) (Fig. 4). Eighteen months after surgery, he had melena with negative findings on both upper and lower gastrointestinal endoscopic examinations. CT scans revealed varices at a portion of the PJ that communicated with a vein of the elevated jejunal limb and the PV (Fig. 5a, b). We selected obliteration of the varices via a trans-portal-venous approach by puncturing the intrahepatic PV. The varices around the PJ disappeared after obliteration using ethanolamine oleate iopamidol (Fig. 5c, d). Follow-up CT at 1 year after this procedure showed no varices at a portion of the PJ (Fig. 6). His melena had disappeared until his death due to a recurrence of PC 17 months after IVR.

## Discussion

Previously, some authors have shown that PD without SV reconstruction could cause LSPH [3, 4]. However, until now, there was no definitive procedure to prevent LSPH after PVR without SV reconstruction because certain LSPH mechanisms after a PD were unclear. Gyoten et al. [5] reported that concomitant splenic artery divisions during operations may attenuate the risk of LSPH. Some authors [6, 7] also advocated that preserving the SV-IMV confluence or reconstruction of the SV-IMV anastomosis would be useful for preventing LSPH. However, Ono [8] and Ismael et al. [9] showed that SV-IMV anastomosis or SV-IMV confluence preservation was insufficient because blood flow from the SV eventually drains into the SMV via the marginal veins of the colon and/or omentum. Findings of our cases support their findings. The present cases showed that preservation of the SV-IMV flow was not useful in preventing LSPH. One possible reason for the formation of varices at a portion of the PJ is the division of communication from the SV to the greater omentum and marginal veins of the transverse and right colon. Because we usually separate the omentum from the colon when we perform a PD, our experiences supported Ono and Ismael’s opinions. Recently, some authors [10, 11] have shown that the preservation of collateral veins prevents LSPH. Considering these findings, preservation of many drainage vein of splenic flow may reduce the risk of LSPH.

For intestinal bleeding from a PJ due to LSPH, several types of procedures can be considered: total pancreatectomy, a splenectomy, obliteration of the varices via a trans-portal-venous approach, and a PSE. Previously, several authors have reported that PSE is an effective treatment for acute gastric bleeding due to LSPH [12, 13]. Our first case suggested that PSE would be effective not only for gastric bleeding but also for bleeding from PJs due to LSPHs. PSEs are also thought to be less invasive than splenectomies. However, PSEs could cause severe postoperative reactions such as splenic abscess, sepsis, and splenic ruptures [14]. Wang et al. [15] reported that splenic infarctions following PSEs could be controlled in 60–70% of patients. However, some recent reports have indicated that a total splenic embolization may be associated with lower rates of splenic abscess formation than partial embolization [16, 17].

In our second case, we selected obliteration of the varices at the PJ via a trans-portal-venous approach because CT scans showed a route from the PV to the varices at a portion of the PJ. This procedure was previously reported by Sakamoto et al. [18] in 2014. This procedure had several benefits that could maintain the splenic arterial flow and approach the varices directly. However, the procedure is highly technical. In our first case, we cannot select obliteration of the varices because the approach to the varices from PV was thought to be difficult due to tortuosity of the vein.

Our cases suggested that IVR could be the first choice for intestinal bleeding at a portion of the PJ due to LSPH. Furthermore, it is assumed that trans-portal-venous approach is first-choice procedure.

## Conclusions

We presented two types of IVR procedures for intestinal bleeding at a portion of the PJ following PD. The obliteration of the varices in PJ could be first choice procedure.

## Abbreviations

CT: Computed tomography
IMV: Inferior mesenteric vein
IVR: Interventional radiology
LSPH: Left-sides portal hypertension
PC: Pancreatic cancer
PD: Pancreaticoduodenectomy
PJ: Pancreaticojejunostomy
PSE: Partial splenic artery embolization
PV: Portal vein
PVR: Portal vein resection
SMV: Superior mesenteric vein
SV: Splenic vein
